# Prognostic Significance of Promoter DNA Hypermethylation of *cysteine dioxygenase 1 (CDO1)* Gene in Primary Breast Cancer

**DOI:** 10.1371/journal.pone.0144862

**Published:** 2016-01-19

**Authors:** Naoko Minatani, Mina Waraya, Keishi Yamashita, Mariko Kikuchi, Hideki Ushiku, Ken Kojo, Akira Ema, Hiroshi Nishimiya, Yoshimasa Kosaka, Hiroshi Katoh, Norihiko Sengoku, Hirokazu Tanino, David Sidransky, Masahiko Watanabe

**Affiliations:** 1 Department of Surgery, Kitasato University School of Medicine, Sagamihara, Kanagawa, Japan; 2 Department of Otolaryngology, Johns Hopkins University, Baltimore, Maryland, United States of America; 3 Department of Surgery, Yamato Municipal Hospital, Yamato, Kanagawa, Japan; King Faisal Specialist Hospital & Research Center, SAUDI ARABIA

## Abstract

Using pharmacological unmasking microarray, we identified promoter DNA methylation of *cysteine dioxygenase 1 (CDO1)* gene in human cancer. In this study, we assessed the clinicopathological significance of *CDO1* methylation in primary breast cancer (BC) with no prior chemotherapy. The *CDO1* DNA methylation was quantified by TaqMan methylation specific PCR (Q-MSP) in 7 BC cell lines and 172 primary BC patients with no prior chemotherapy. Promoter DNA of the *CDO1* gene was hypermethylated in 6 BC cell lines except SK-BR3, and *CDO1* gene expression was all silenced at mRNA level in the 7 BC cell lines. Quantification of *CDO1* methylation was developed using Q-MSP, and assessed in primary BC. Among the clinicopathologic factors, *CDO1* methylation level was not statistically significantly associated with any prognostic factors. The log-rank plot analysis elucidated that the higher methylation the tumors harbored, the poorer prognosis the patients exhibited. Using the median value of 58.0 as a cut-off one, disease specific survival in BC patients with *CDO1* hypermethylation showed significantly poorer prognosis than those with hypomethylation (p = 0.004). Multivariate Cox proportional hazards model identified that *CDO1* hypermethylation was prognostic factor as well as Ki-67 and hormone receptor status. The most intriguingly, *CDO1* hypermethylation was of robust prognostic relevance in triple negative BC (p = 0.007). Promoter DNA methylation of *CDO1* gene was robust prognostic indicator in primary BC patients with no prior chemotherapy. Prognostic relevance of the *CDO1* promoter DNA methylation is worthy of being paid attention in triple negative BC cancer.

## Introduction

Breast cancer (BC) is the second most common malignancy worldwide. According to GLOBOCAN 2012 statistics, nearly 1.7 million women were estimated as new cases (25% of all cancers) with the fifth leading cause of cancer-related deaths (522,000 deaths in 2012). BC is classified into 4 definite entities which were composed of luminal A, luminal B, HER2, and triple negative BC according to hormone receptors and HER2 expression [1–3]. Triple negative BC (TNBC), accounting for about 15% of BC and characterized by negativity for Estrogen Receptor (ER), Progesterone Receptor (PR), and HER2, is associated with aggressive histology, poor prognosis, and unresponsiveness to the usual endocrine therapies [4–6]. Biomarker selection will be thus important, in order to identify patients, especially, with TNBC who were the most likely to benefit from selected treatments.

BC is a genetic disease, and recent advances in molecular biology have revealed recurrent genetic and/or epigenetic alterations [7] Epigenetic gene silencing of the tumor suppresser genes through promoter DNA hypermethylation is a common feature in human cancers, whereas cancer specific methylation is rather a rare event [8–11]. We have developed pharmacologic reversal of epigenetic silencing and uncovered a myriad of transcriptionally repressed genes in human cancers [12–15]. Using this technique, we have identified novel tumor suppressor gene candidates including cysteine dioxygenase type 1 (*CDO1*) gene in primary BC [12].

The human *CDO1* gene is located on chromosome 5q23 [12], which was reported to be likely associated with distant metastasis of BC [16]. *CDO1* gene is a non-heme structured, iron-containing metalloenzyme involved in conversion of the cysteine to cysteine sulfinic acid (CSA) [17–19], while it may promote apoptosis by increasing reactive oxygen species (ROS) through suppression of glutathione (GSH) generation [20] (S1 Fig). Jeschke et al demonstrated that *CDO1* gene is significantly associated with anthracyclin sensibility, and promoter DNA hypermethylation of *CDO1* gene relates to negative prognostic outcome in BC patients who performed preoperative anthracyclin therapy [21]. In this study, we for the first time investigated clinicopathologic and prognostic relevance of promoter DNA methylation of *CDO1* gene in BC with no preoperative chemotherapy.

## Materials and Methods

### BC cell lines and tissue samples

The BC cell lines, SK-BR3, YMB1, CRL, and MDA-MB231 cells were kindly provided from the Kyusyu University (Oita, Japan) They were obtained by the Cell Resource Center for Biomedical Research Institute of Development, Aging and Cancer, Tohoku University (Sendai, Japan) and HMEC (provided by LifeLine) [22][23]. The other BC cell lines, YMB1E and colorectal cancer (CRC) cell line DLD1 [15] were provided from the Cell Resource Center for Biomedical Research Institute of Development, Aging and Cancer, Tohoku University (Sendai, Japan). Two other BC cell lines, MCF7 and MDA-MB453, or the hepatocellular carcinoma (HCC) cell line HepG2 [15] were purchased from RIKEN BioResource Centre (Ibaraki, Japan). MCF7, SK-BR3, YMB1, CRL, and YMB1E were maintained in RPMI 1640 Medium (GIBCO, Carlsbad, CA) and MDA-MB453 was maintained in L-15 (GIBCO) and MDA-MB453 was maintained in DMEM (Sigma Aldrich, USA), containing 10% fetal bovine serum and Penicillin-Streptomycin (GIBCO). The cell lines were cultured at 4–5 passaging stage to examined.

We recruited 253 primary BC patients with no prior chemotherapy who underwent surgical resection of the primary tumors at the Kitasato University Hospital between January 1, 1995 and December 31, 1999 [24]. Of the 253 patients, we extracted DNA from the formalin-fixed, paraffin embedded (FFPE) primary tumor tissues of the 172 BC patients who agreed to use pathological specimens. Background of the 172 BC patients were shown in S1 Table.

TNM classification was made according to the latest 7^th^ edition of the Union for International Cancer Control (UICC). All tissue samples were collected at the Kitasato University Hospital, and written informed consent was obtained from all patients and healthy donors before sample collection. The present study was approved by the Ethics Committee of Kitasato University.

### Bisulfite Treatment of DNA and Sequencing Analysis

Genomic DNA of FFPE and cell lines were extracted using QIAamp DNA FFPE Tissue Kit and QIAamp DNA Mini Kit (QIAGEN Sciences, Maryland, MD). Bisulfite treatment was done by using a Methylation-Gold Kit (QIAGEN). Primer sequences for the genes of interest were designed to recognize this DNA alterations (S2 Table). The primer products were sequenced using a Big Dye^®^ Terminator v3.1 Cycle Sequencing Kit (Applied Biosystems, Foster City, CA).

### Quantitative-Methylation-specific PCR (Q-MSP)

TaqMan methylation specific PCR (Q-MSP) was carried out using iQ Supermix (Bio-Rad) in triplicate on the C1000 Touch^TM^ Thermal Cycler CFX96 Real Time System (Bio-Rad). PCR conditions and the primer sequences are provided in S2 Table. Serial dilutions of bisulfite modified DNA from CRC cell line DLD1 was used to construct the calibration curve on each plate as methylation positive control, and HCC cell line HepG2 was used as negative control, respectively [15]. The methylation value (designated as TaqMeth Value as previously described [12]) was defined by a ratio of amplified signal value of methylated *CDO1* normalized to β-actin and then multiplied by 100.

### RNA purification and reverse transcription-polymerase chain reaction (RT-PCR)

Total RNA from cell lines and primary tumors were extracted using Rneasy Mini Kit and RNeasy FFPE Kit (Qiagen). Reverse-transcribed with SuperScript III reverse transcriptase kit (Invitrogen). Primers sequences are also included in S2 Table. RT-PCR was performed, and the PCR products were separated on 1.5–2.0% agarose gel, then visualized by ethidium bromide staining. β-actin was used as an internal control.

### Statistical analysis

Student’s *t*-test was used for continuous variables, and χ^2^ test was used for categorical variables. Clinicopathologic characteristics and follow up data were analyzed in terms of disease specific survival (DSS). The follow up time was calculated from the date of surgery to death, and patients with other disease deaths were defined as censored ones. DSS was calculated by Kaplan-Meier method, and survival differences were assessed in the log-rank test. Variables suggested to be prognostic factors on univariate analysis (P<0.05) were subjected to multivariate analysis using a Cox proportional-hazards model. P-value <0.05 was considered to indicate statistical significance. All statistical analyses were conducted with SAS software package (JMP Pro11, SAS Institute, Cary, NC).

## Results

### *CDO1* promoter methylation is frequent in BC cell lines

**Fig 1:**
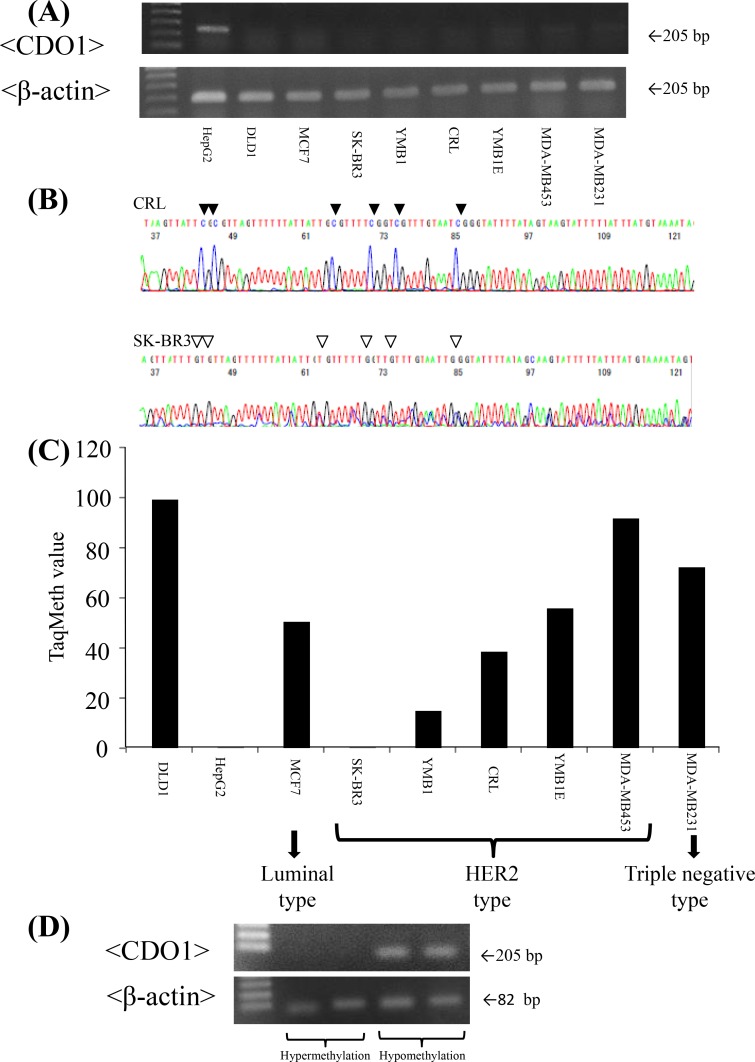
*CDO1* methylation and expression in BC cell line. **A,** *CDO1* mRNA expression in BC cell lines was assessed by semi-quantitative reverse transcribed PCR (RT-PCR). B, Representative direct bisulfite sequence results in CRL cells (methylation) and SK-BR3 cells (unmethylation). C, *CDO1* mRNA expression in BC cell lines was assessed by Q-MSP. (D) *CDO1* mRNA expression in BC tissues was assessed by RT-PCR.

We initially examined 7 BC cell lines to know expression status of *CDO1* gene. *CDO1* gene expression was barely detected at mRNA level in all BC cell lines as compared to the HepG2 (Fig 1A).

We then examined promoter DNA methylation status of the *CDO1* gene in all the 7 BC cell lines by bisulfite treatment followed by direct sequencing and Q-MSP analysis. Promoter DNA of the *CDO1* gene was proved to be completely methylated in cytosine residues of CpG islands in 6 BC cell lines except SK-BR3 (Fig 1B and 1C). This finding indicated that promoter DNA hypermethylation of the *CDO1* gene may at least partially explain the mechanism of gene silencing of *CDO1* at mRNA level in a large portion of BC cell lines.

We also examined promoter DNA methylation status of the *CDO1* gene by Q-MSP in BC cell lines, and confirmed HepG2 and SK-BR3 cells to be completely unmethylated as results of direct sequence. DNA methylation level of the *CDO1* gene is unlikely to be associated with BC subtypes (Fig 1C).

### Expression of *CDO1* transcripts in BC tissues

We examined the expression status of *CDO1* transcripts for the primary tumors tissues in 10 cases with hypermethylation group and 10 cases with hypomethylation group, respectively by semi-quantitative RT-PCR. As a result, all in 10 cases had expression of β actin in hypomethylation group and 4 of 10 cases had expression of *CDO1*. In hypermethylation group, only 3 of 10 cases showed expression of β actin and 2 of 3 cases that showed expression of β actin had expression of *CDO1* (representative cases were shown in Fig 1D).

### *CDO1* promoter methylation level and its correlation with clinicopathologic factors in primary BC tissues

**Fig 2:**
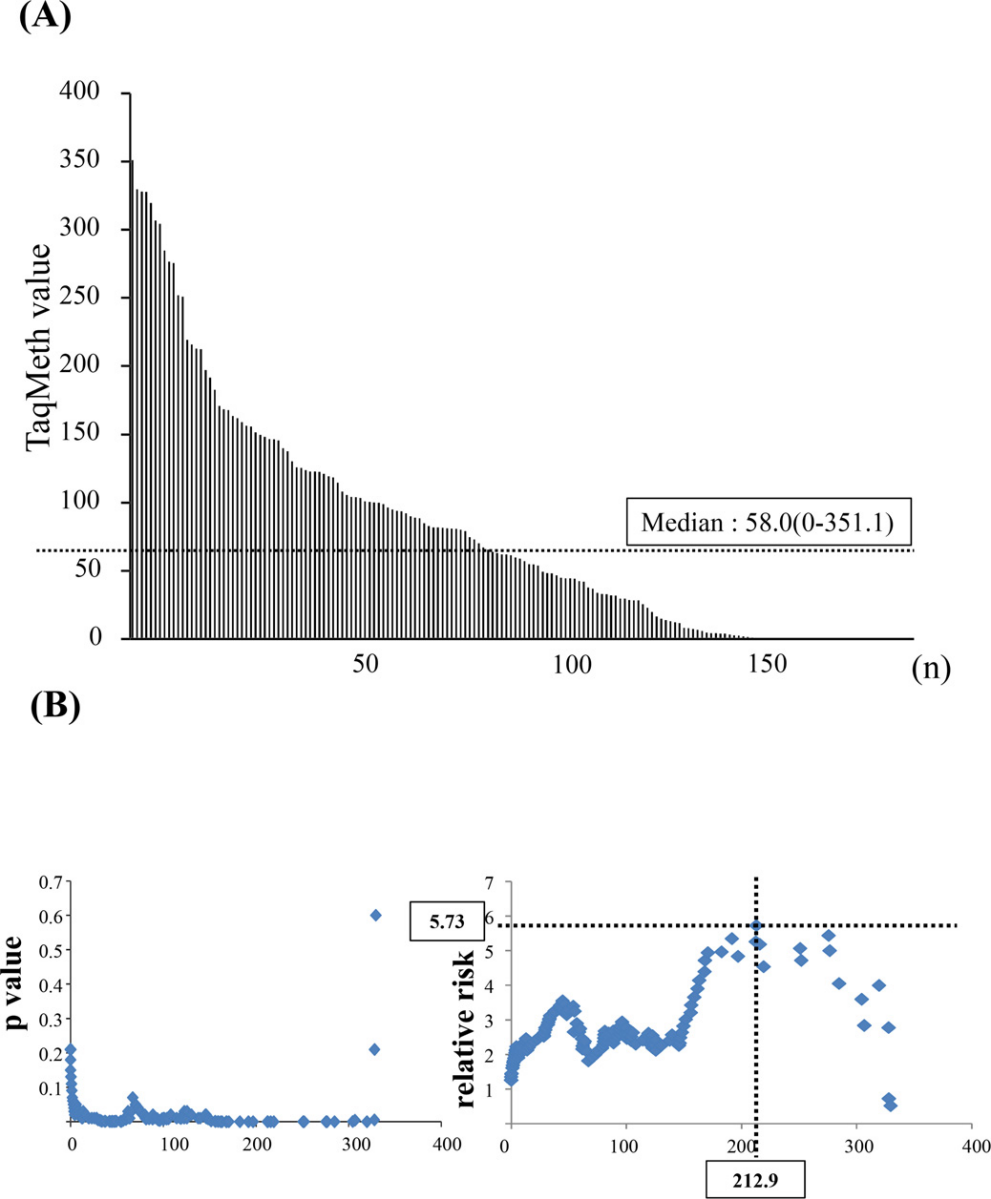
Quantitative assessment of *CDO1* methylation in primary BC tissues. **A,** TaqMeth value of 172 BC tissues.Median is median 58.0 (0–351.1). B, p value and relative risk were plotted according to the Log rank test. Note that the p value is constantly below 0.05, suggesting that the higher methylation value of *CDO1* gene is, poorer prognosis the patients exhibited in primary BC.

Next, to clarify the clinical significance of the methylation level of the *CDO1* gene, Q-MSP assessment of the BC tumor tissues was also performed in 172 primary BC. The median TaqMeth value was 58.0, ranging from 0 to 351.1 in primary BC tumor tissues (Fig 2A). Correlation of each clinicopathologic factor to quantitative methylation value of the*CDO1* gene in primary BC tumor tissues was compared by Student *t*-test. Although there was no statistical difference between *CDO1* promoter methylation level to size of tumor (pT factor), lymph node metastasis (pN factor), UICC staging system, hormone receptor status, and Ki-67 status, but it tended toward with HER2 (p = 0.07) (S2 Fig). There was no significant difference between CDO1 promoter methylation and the histological type (p = 0.84).

### Univariate Prognostic Analysis including *CDO1* promoter DNA methylation status in primary BC tissues

We further investigated whether the *CDO1* TaqMeth value was able to predict prognostic outcomes of primary BC. A Kaplan-Meier curve for the 172 patients was constructed to analyse survival discrepancies according to *CDO1* TaqMeth values above or below each cut-off value by the log rank plot method, and when log-rank p value for the DSS remained almost constantly below 0.05 (significant) by almost all cut-off value (Fig 2B). This result indicated that the higher *CDO1* TaqMeth value was, the worse the prognosis was, and *CDO1* TaqMeth value was the ideal prognostic marker.

**Fig 3:**
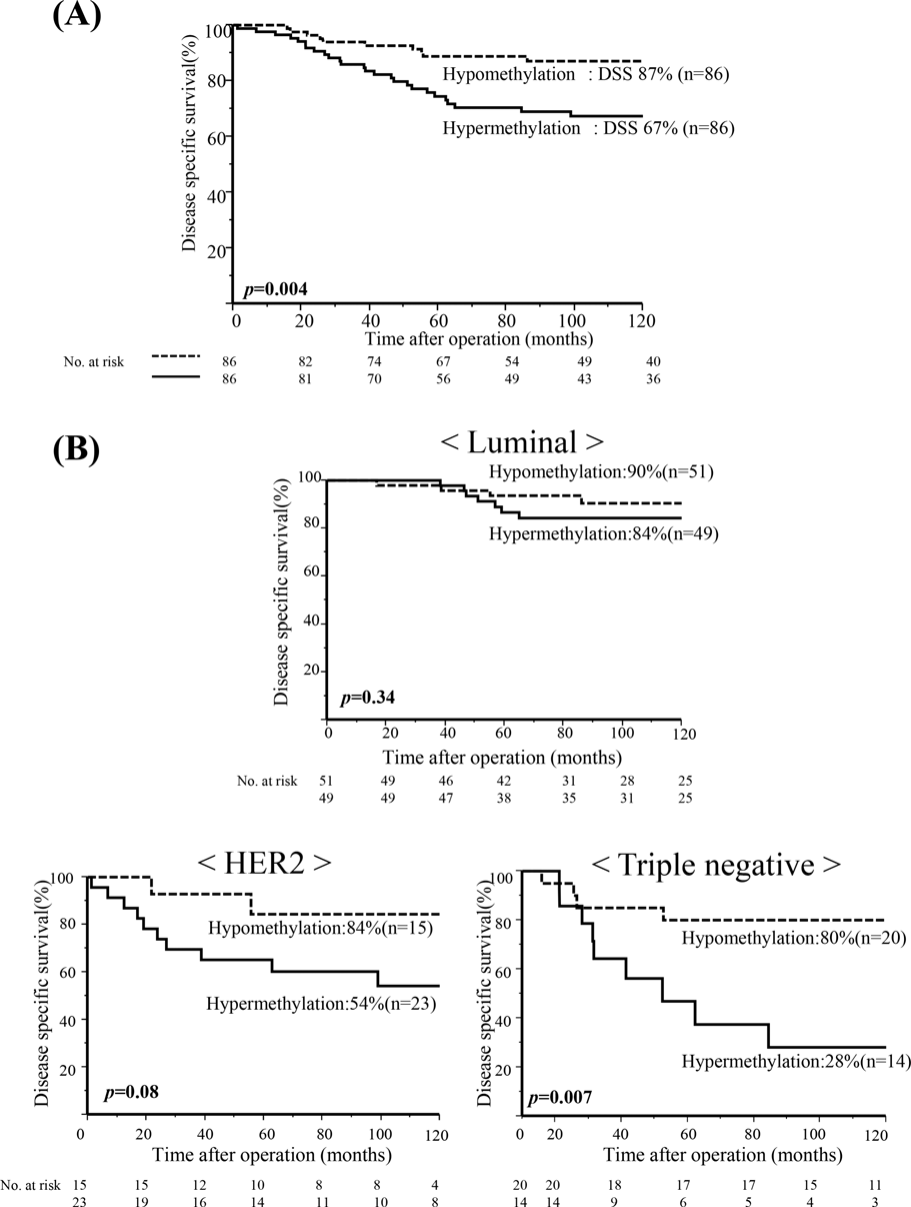
*CDO1* gene methylation and prognosis according to BC subtypes. A, Kaplan-Meier curve for DSS is shown in total primary BC. The cut-off value was median value (58). Patients with *CDO1* hypermethylation exhibited significantly poorer prognosis than those with *CDO1* hypomethylation in primary BC (p = 0.004). B, Kaplan-Meier curves for DSS are shown according to subtypes.

We therefore defined cut off value of *CDO1* TaqMeth value as 58.0 that was the median of *CDO1* TaqMeth value for DSS, where DSS in hypermethylation group was 67% (n = 86), and that in hypomethylation group was 87% (n = 86), and the prognostic difference was robust (p = 0.004)(Fig 3A and Table 1). In other words, hypermethylation group exhibited significantly poorer prognosis than hypomethylation group.

**Table 1:** Univariate and multivariate analysis for disease specific survival (DSS).

	DSS					
	Univariate analysis			Multivariate analysis		
Factors	patient number	DSS (%)	*p*	HR*	95%CI	*p*
Age			0.03			
51>	86	70				
51<	86	83				
Gender			0.61			
Female	171	77				
Male	1	100				
pT factor			<0.0001			
T1,2	154	82				
T3,4	18	35				
pN			0.002			
negative	88	87				
positive	84	67				
pStage (7^th^ UICC)			<0.0001			
1	51	87		Reference		
2	76	85		1.0	0.4–3.0	1.0
3	45	51		2.8	1.2–7.9	0.02
Ki-67			<0.0001	5.2	2.7–10.2	<0.0001
negative	141	87				
positive	31	38				
Hormone receptor			0.0006	3.2	1.4–7.5	0.006
negative	54	62				
positive	118	84				
TaqMeth value			0.004	2.4	1.2–5.4	0.01
58<	86	87				
58>	86	67				
HER2 receptor			0.03	1.4	0.6–3.1	0.45
positive	38	65				
negative	134	80				
Subtype			0.0004			
Luminal type	100	88				
HER2 type	38	65				
Triple negative type	34	60				

* HR: Hazard Ratio

The clinicopathologic factors related to prognosis were then examined in the multivariate analysis. As a result, Ki-67 positive (p = <0.0001), hormone receptor negative (p = 0.006), and high *CDO1* TaqMeth value (p = 0.01) were independent prognostic factors profoundly related to DSS in primary BC (Table 1).

### Correlation of clinicopathologic factors to promoter DNA methylation status that was determined according to median *CDO1* TaqMeth value in primary BC

Correlation of clinicopathologic factors in primary BC with promoter DNA methylation status that was determined according to median *CDO1* TaqMeth value by χ^2^ test (Table 2). BC patients with aggressive lymph node metastasis more frequently exhibited promoter DNA hypermethylation of *CDO1* than those with modest lymph node metastasis (p = 0.01). Reflected on this finding with regard to lymph node metastasis, *CDO1* promoter DNA hypermethylation is also significantly associated with stage (p = 0.02).

**Table 2:** Correlation of clinicopathologic characterestics and *CDO1* methylation.

	*CDO1* TaqMeth value				
	low (< 58.0)		high (> 58.0)		
	(n = 86)		(n = 86)		
Factors	No.	%	No.	%	*p*
Age (median)	51.1 (22–77)		51.1 (29–84)		0.48
Operation method					0.31
Lumpectomy	28	56.0	22	44.0	
Mastectomy	58	47.5	64	52.5	
pT factor*					0.29
T1	47	57.3	35	42.7	
T2	30	41.7	42	58.3	
T3	7	50.0	7	50.0	
T4	2	50.0	2	50.0	
pN factor*					0.01
pN0	52	59.1	36	40.9	
pN1	21	50	21	50.0	
pN2	5	21.7	18	78.3	
pN3	8	42.1	11	57.9	
pStage*					0.02
1	31	60.8	20	39.2	
2	40	52.6	36	47.4	
3	15	33.3	30	66.7	
Pathological type					0.55
Invasive ductal carcinoma	79	49.4	81	50.6	
Others	7	58.3	5	41.7	
Hormonal receptor (IHC)					0.32
Positive	56	47.5	62	52.5	
Negative	30	55.6	24	44.4	
HER 2 (IHC)					0.14
Positive	15	39.5	23	60.5	
Negative	71	53.0	63	47.0	
Ki-67 (IHC)					0.32
Positive	13	41.9	18	58.1	
Negative	73	51.8	68	48.2	
Subtype					0.25
Luminal type	51	51.0	49	49.0	
HER2 type	15	39.5	23	60.5	
Triple negative type	20	58.8	14	41.2	

*: 7th edition of the Union for International Cancer Control (UICC)

### Prognostic relevance of *CDO1* TaqMeth value in BC subtypes

We also examined DSS of individual subtypes (Luminal type, HER2 type, and TN type) according to *CDO1* TaqMeth value, and revealed that hypermethylation group showed poorer prognosis than hypomethylation group in any subtypes. Especially, hypermethylation group was extra-ordinarily poorer prognosis in TNBC (p = 0.007) (Fig 3B).

TNBC showed more frequent postoperative recurrence rate (44.1%) as compared with other cases (29.7%) (p = 0.11), even though 70.6% of TNBC underwent postoperative adjuvant therapy as compared with 32.6% of total cases. TNBC included progressive cases with regard to stage (stage II: 50.0%, stage III: 26.5%) similarly with total cases (stage II: 44.2%, stage III: 26.1%). Furthermore, there were more Ki-67 positive BC patients in TNBC (32.3%) in a comparison with those in other cases (14.5%) (p = 0.02).

## Discussion

We recently performed prognostic analysis including 4 subtypes (Luminal A, Luminal B, HER2, and Triple negative) in primary BC patients, and proved that Ki-67 has a great potential as a prognostic biomarker [24]. St.Gallen International Breast Cancer Conference recently adopted Ki-67 for subtype classification of BC [1]. In this study, we elucidated that *CDO1* promoter hypermethylation was strongly related to poor prognosis as well as Ki-67 in primary BC.

*CDO1* catalyzes the oxidation of cysteine to cysteine sulfinic acid (CSA) [17]. CSA inhibits pyruvate dehydrogenase (PDH) activity in mitochondria [25] and subsequent activation of citric acid cycle and generation of ATP through the electron transport chain (cellular respiration) [26]. As a result, CSA suppresses an efflux of H+ from mitochondria to intracellular compartment of the cells, leading to sustained mitochondrial membrane potential [26]. Such a cellular status inhibits apoptosis and therefore tumorigenesis is enhanced in glioma cells, where *CDO1* or CSA is augmented [26].

On the other hand, we have recently identified *CDO1* as genes methylated specifically in human cancers after developing algorithm utilizing pharmacological unmasking microarray (PUM) [12–14] as well as others [21] [27], suggesting that *CDO1* plays a tumor suppressive role in human carcinogenesis. Promoter DNA of the *CDO1* gene was frequently methylated in breast, esophagus, lung, bladder, gastric, and colorectal cancers [12]. It is well known that such cancer-prone methylation is characteristic of tumor suppressor gene, and these findings are opposite to the theory recognized in glioblastoma. It has been therefore thought that clinical significance of *CDO1* gene expression depends on the organs [26].

There have been several reports describing clinical significance of *CDO1* gene promoter DNA methylation in primary BC. Dietrich et al. demonstrated promoter DNA hypermethylation of *CDO1* gene in primary BC and showed clinical potential as a predictor of distant metastasis in primary BC patients with lymph node metastasis[27]. In this report, they actually presented data that *CDO1* gene methylation actually exhibited poor prognosis. Jeschke also proved that promoter DNA methylation of *CDO1* gene is significantly correlated with tumor progression and, intriguingly, prognostic relevance was found in primary BC patients who were treated by anthracycline. They also presented data that *CDO1* induced reactive oxygen species (ROS) in BC cells, and this biological traits could explain the mechanism of tumor growth retardation and sensitivity to anthracycline [21]. Unmethylated *CDO1* status of the primary BC tissues further revealed somatic missense mutations in 17% of these tumors [21]. ROS production and augmentation of anticancer drug sensitivity are not found in such *CDO1* mutant transfectants. In other words, such a mutation also represented loss of function of the tumor suppressor gene in primary BC.

In this study, we investigated promoter DNA methylation status of the *CDO1* gene in 172 primary BC tumor tissues, and strong association of *CDO1* gene promoter DNA methylation with poor prognosis was shown in primary BC patients, especially for triple negative BC (TNBC). Because we used the tumor tissues in which DNA status was not modified by neoadjuvant chemotherapy (NAC), the prognostic relevance we proposed in this current study may represent natural clinical curse of the primary BC. *CDO1* methylation did not have significant correlation to individual subtypes and other prognostic factors. Reflected on this finding, *CDO1* methylation could be an independent prognostic factor in the multivariate prognostic analysis. More intriguingly, the higher *CDO1* TaqMeth value was, the worse the prognosis was, in almost all cut-off value. Therefore, *CDO1* gene methylation status is regarded as an ideal prognostic indicator of the primary BC.

TNBC is well known to show poor prognosis, and it is specifically expressed for lactose dehydrogenease (LDH) [28], where it enhanced the Warburg effect. This finding suggested that TNBC is dependent on the Warburg effect in terms of ATP generation [29–30]. TNBC cell line was actually decreased in tumorigenesis by knockdown of LDH [28]. *CDO1* methylation was not found to be specific for TNBC, but prognosis was especially poor in primary TNBC with a *CDO1* hypermethylation. The anaerobic metabolism through LDH for ATP generation, which is supposed to be affect citric acid cycle, secures cell viability of TNBC cells, and *CDO1* promoter DNA hypermethylation with its reduced expression may affect TNBC cysteine metabolism so much.

Cysteine biology was recently focused on cancer stem cell biology. CD44 variant (CD44v) interacts with xCT, a glutamate-cystine transporter, and controls the intracellular level of reduced glutathione (GSH) [31]. Human cancer stem-like cells with a high level of CD44 expression showed an enhanced capacity for GSH synthesis and defense against reactive oxygen species (ROS). Ablation of CD44 induced loss of xCT from the cell surface and suppressed tumor growth. xCT is actually expressed on one-third of TNBC, and xCT inhibition decreases tumor growth of BC [32–33]. Cysteine metabolism modified by aberrant expression of *CDO1* gene is thus considered to play an important role in cancer cell stemness in TNBC.

Limitations; in this study, there is no patient who underwent preoperative neoadjuvant chemotherapy (NAC) in TNBC, however NAC is becoming standard treatment now. It will be necessary to examine relation of *CDO1* methylation status to clinicopathological factors including prognosis in TNBC patients who undertook NAC in the near future. On the other hand, treatment strategy has not changed so much in the luminal type. The methylation of *CDO1* gene was not, however, a significant prognostic factor in the luminal type, putatively because it exhibited excellent prognosis in nature. As for HER2 type, hypermethylation group of *CDO1* gene tended to show poorer prognosis as compared to hypomethylation group, and it could be a prognostic predictor. Clinical use of trastuzumab, anti-HER2 antibody, was approved from 2001 in Japan and did not use trastuzumab in this current study. It will be important to investigate as prognostic relevance of methylation status of the*CDO1* gene in HER2 type BC patients who underwent trastuzumab treatment.

In conclusion, we demonstrated that methylation of the *CDO1* gene promoter could be strong prognostic indicator in primary BC without preoperative treatment. However, prognostic significance of methylation of *CDO1* gene remains obscure at present, especially in HER2 and TNBC patients with the latest treatments. Clinical use of *CDO1* gene methylation status in BC clinics should be done after such validation.

## Limitation

In this current study, we did not detect *β actin* gene expression in all tested cases at mRNA level, putatively because the tested specimens were formalin fixed, limited to the very early cases in 1990's. *CDO1* mRNA expression is not necessarily very accurately assessed for all tested cases. Such preliminary results with regard to expression assessment at mRNA level could represent consistent outcomes of promoter DNA methylation in primary tumor tissues.

## Conclusions

Promoter DNA methylation of *CDO1* gene was robust prognostic indicator in primary BC patients with no prior chemotherapy. Prognostic relevance of the *CDO1* promoter DNA methylation is worthy of being paid attention in triple negative BC cancer.

## Supporting Information

S1 Fig Cysteine metabolism in cancer cells. (TIF)

S2 Fig Correlation of *CDO1* methylation values to clinicopathological factors in primary BC. The methylation of *CDO1* gene was not related with any prognostic factors such as stage and subtypes.
(TIF)

S1 Table Clinicopathologic characteristics of the 172 patients. (XLSX)

S2 Table PCR production and sequence of primers and fluorescent probe. (XLSX)
