# Supplementary figures and images for: Prognostic Significance of Promoter DNA Hypermethylation of cysteine dioxygenase 1 (CDO1) Gene in Primary Breast Cancer
Source: PLoS One. 2016 Jan 19;11(1):e0144862. doi: 10.1371/journal.pone.0144862 (PMC4718689; doi:10.1371/journal.pone.0144862)

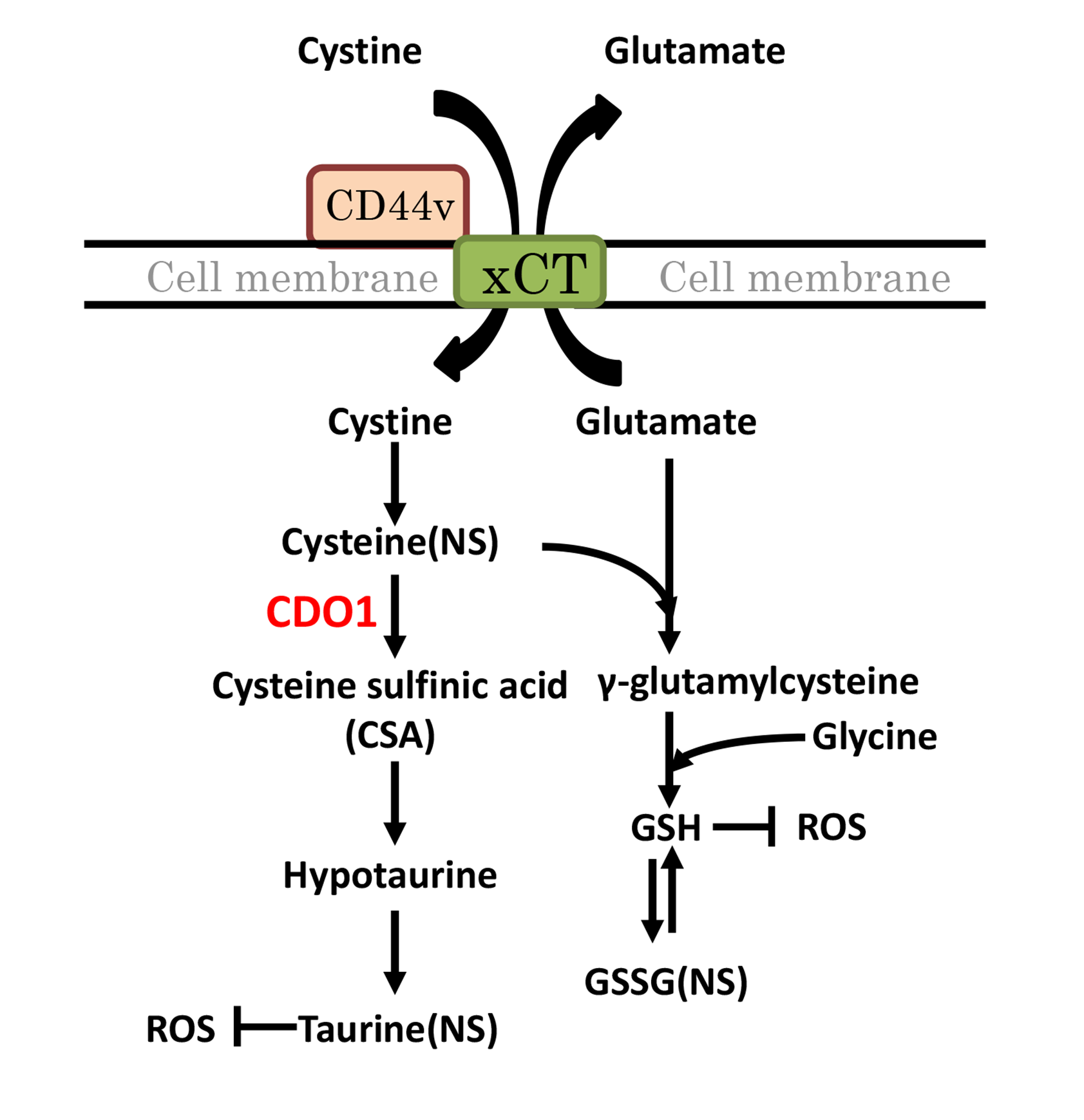

Supplement: S1 Fig — (TIF) [file pone.0144862.s001.tif]

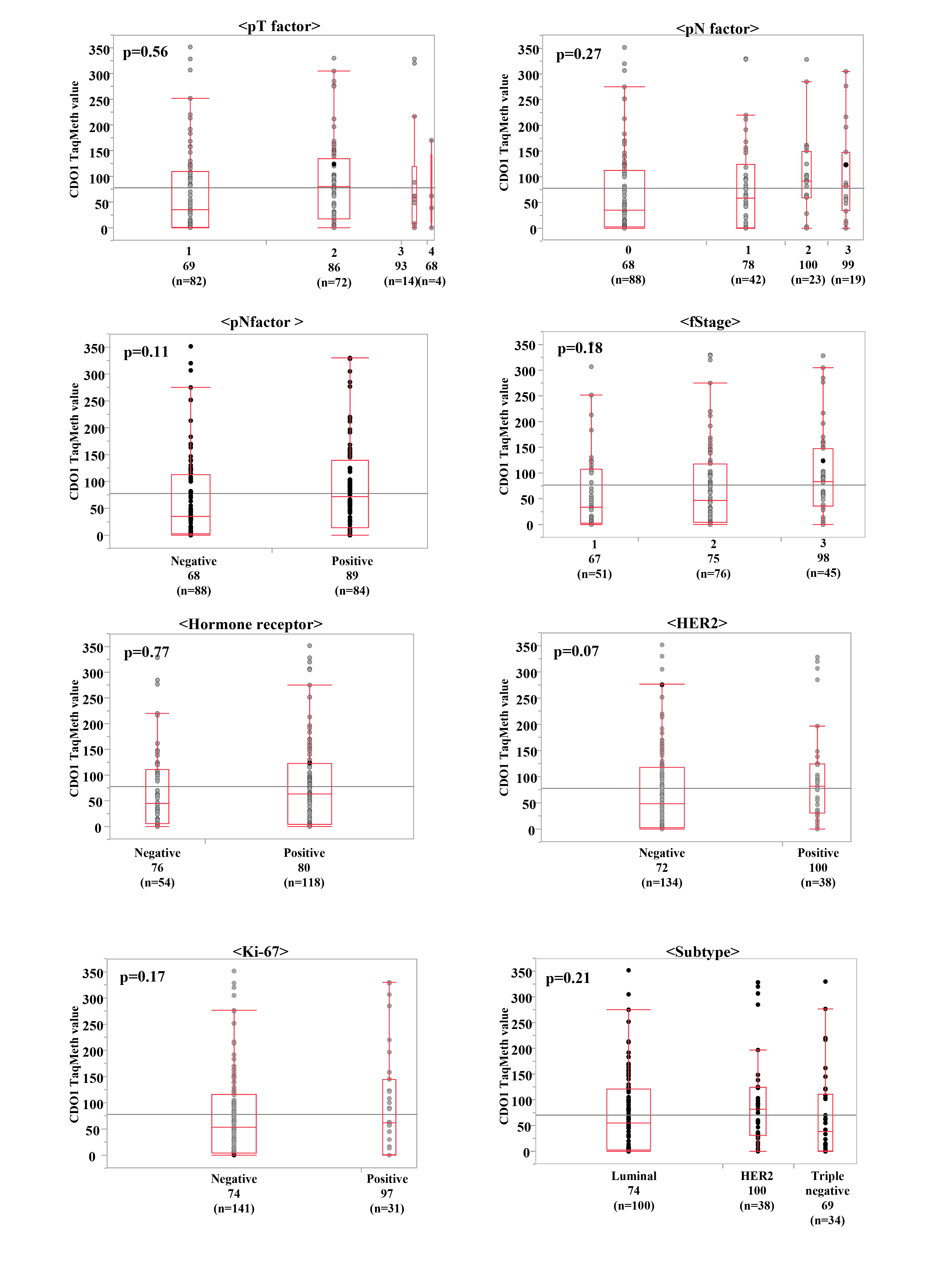

Supplement: S2 Fig — The methylation of CDO1 gene was not related with any prognostic factors such as stage and subtypes. (TIF) [file pone.0144862.s002.tif]
